# Rotational and translational osteotomy for treatment of severe deformity in hypophosphatemic rickets: A case report

**DOI:** 10.1097/MD.0000000000018425

**Published:** 2020-01-17

**Authors:** Jin Li, Saroj Rai, Renhao Ze, Xin Tang, Ruikang Liu, Pan Hong

**Affiliations:** a Department of Orthopaedic Surgery, Union Hospital, Tongji Medical College, Huazhong University of Science and Technology, Wuhan, China; b Department of Orthopaedics and Trauma Surgery, National Trauma Center, National Academy of Medical Sciences, Mahankal, Kathmandu, Nepal; c First School of Clinical Medicine, Tongji Medical College, Huazhong University of Science and Technology, Wuhan, China.

**Keywords:** hypophosphatemic rickets, rotational and translational osteotomy, surgical intervention

## Abstract

**Rationale:**

Hypophosphatemic rickets (HR) is a rare hereditary disease characterized by hypophosphatemia, defects in bone mineralization, and rickets, and surgical intervention is warranted for the patient of severe skeletal deformity.

**Patient concerns:**

Here we report a case of an 11-year-old boy who presented with severe varus deformities of the bilateral lower extremities and was associated with uncoordinated gait with multiple unintentional falls onto ground resulting in fractures of lower extremities.

**Diagnoses:**

He was diagnosed as HR caused by genetic mutations in the phosphate-regulating endopeptidase homologue. Based on his family history and laboratory tests, including high serum alkaline phosphatase, high urinary phosphorus, hypophosphatemia, and normal serum calcium level, the patient was diagnosed with this disorder.

**Interventions:**

Rotational and translational osteotomy was performed to redress the severe varus deformity and readjust the malalignment of the lower extremity.

**Outcomes:**

Right after the surgery, the alignment in the left lower extremity was readjusted, and his appearance seemed normal. Combined with rehabilitation and pharmacological intervention, including oral intake of phosphate and alphacalcidol, the bone healed uneventfully. After the second surgery of a similar procedure on the right femur, the patient was able to walk almost like a normal teenager.

**Lessons:**

This case proposed a novel technique to treat severe varus or valgus deformity of the lower extremity. HR is a rare disease, and it is important to stress its recognition to avoid delay of diagnosis and surgical intervention if necessary.

## 1 Introduction

Hypophosphatemic rickets (HR) is a type of hereditary rickets characterized by hypophosphatemia secondary to urinary phosphate loss and defective bone mineralization. X-linked dominant hypophosphatemic rickets (XLHR) is the most common form and is consisted of 80% of HR.^[1]^ Patients of HR is manifested as vitamin D-resistant rickets; however, they usually do not have hypocalcemia, muscle atrophy, and tetany. Individuals of XLHR frequently present following features: short stature, lower extremity deformity, pain, impairment of hearing, rickets, and osteomalacia.^[2,3]^ Radiographic features of rickets includes generalized osteopenia, widened and irregular physes, and cupped and flared metaphyses.

The biochemical array of HR is that of low serum phosphate, increased urinary phosphate, elevated alkaline phosphatase level, and low or low–normal circulating 1,25(OH)_2_Vit-D. Parathyroid hormone level is usually normal on presentation, but demands monitoring throughout growth.^[1]^

Genu varum and/or genu valgum have been reported as the main clinical features in the vast majority of patients with XLHR.^[4]^ Al Kaissi et al^[5]^ advocated corrective surgery at an early stage of deformity in order to avoid prolonged corrective operations, particularly in patients with severe deformities.^[6]^ However, we report a delayed case of HR with severe varus deformity in lower extremity in this case report. The complexity of this delayed case is elucidated in the discussion part.

## 2 Case report

All procedures performed in this study were performed in accordance with the ethical standards of the national research committee and the 1964 Helsinki declaration and its later amendments or comparable ethical standards. The patient's legal guardian provided informed consent for publication of the case.

**Figure 1:**
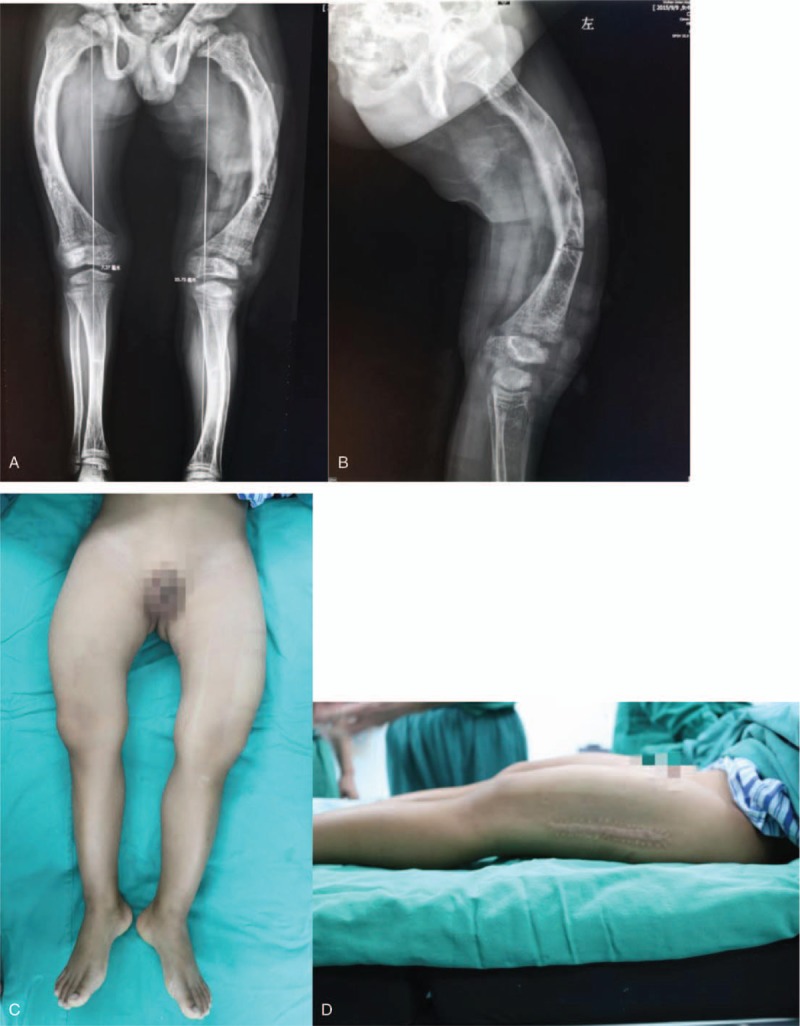
(A) Full-length lower extremity radiograph of 11-year-old boy of left distal femoral fracture; (B) lateral view of femur; (C) appearance of lower extremities on supine position; and (d) lateral view of thigh.

An 11-year-old boy presented with persistent pain in the left thigh after an accidental fall was admitted in our institution in September 2015. Radiography revealed a fracture line at the distal third of the femur without significant displacement (Fig. 1), and genu varum with severe femoral bowing was noticeable.

Physical examinations revealed tenderness at the distal left thigh, and old incision scars about 14 cm on the lateral skin of diaphyseal femur. The genu varum was prominent when the patient was on supine position. The length of lower extremity was equal. There were no cafe au lait spots on the trunk, and no blue sclera. According to the patient, he was unable to walk normally but waddle before this injury. The flexion and extension of hip, knee, and ankle joint seemed within normal range. The patellae moved within normal trajectory without subluxation. Limb muscle tone and physiological reflex were normal, and no pathological reflex was found.

Laboratory findings before operation were as follows: high serum alkaline phosphatase (215 U/L), normal serum calcium (2.5 mmol/L), and low serum phosphate level (1.0 mmol/L).

The patient sustained femoral fractures (2 times on the left, 1 time on the right) 3 times in the past 3 years, and open reduction and internal fixation with plating was performed on the left femur. The plate was removed approximately 6 months before his admission to our institution. The plate was removed after uneventful healing, but the deformity remained as it was. Details were reported on physical examinations.

As for family history, his mother and sister presented with obvious genu varum and hyperextension of the knee joint. The height of his mother was approximately 120 cm.

Based on the radiographic features, clinical manifestations, laboratory results, and family history, the condition was diagnosed as left femoral shaft fracture secondary to HR with genu varum. Although the diagnosis of the disease was pretty straight and simple, the management strategy remained tricky.

**Figure 2:**
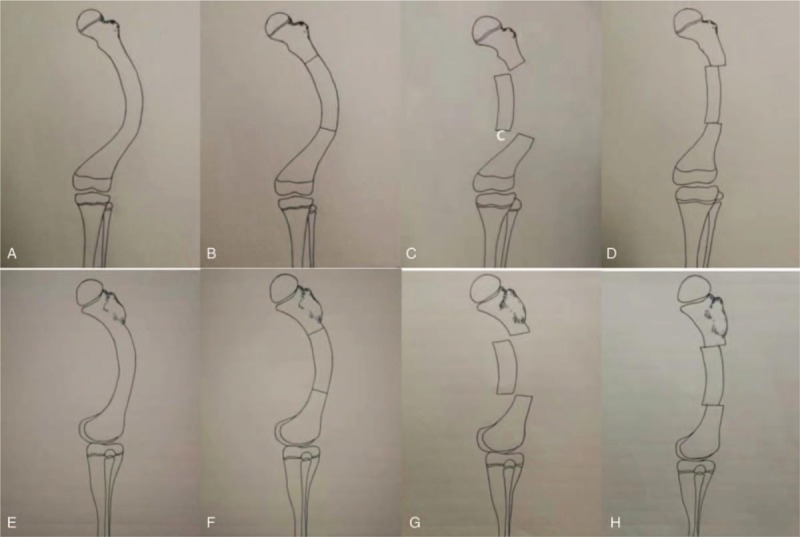
(A–D) Illustration of anterior-posterior view of femur during the operation and (E–H) illustration of lateral view of femur during the operation.

The condition was quite severe with multiplane deformity; only the proximal and distal femoral osteotomy might not be sufficient to redress this malalignment and femoral bowing. Since the patient was a teenager, multiple osteotomies with interlocked nail was also not a feasible option. In this case, external fixators such as Taylor Spatial Frame (TSF) or Ilizarov device might be a powerful tool that allows fine-tuning of the alignment postoperatively. We used a rotational and translational osteotomy to redress its malalignment and femoral bowing, followed by fixation with a Proximal Humerus Internal Locking System (PHILOS) plate (Beijing Libeier Bio-engineering Institute Co Ltd, Beijing City, China) (Fig. 2).

**Figure 3:**
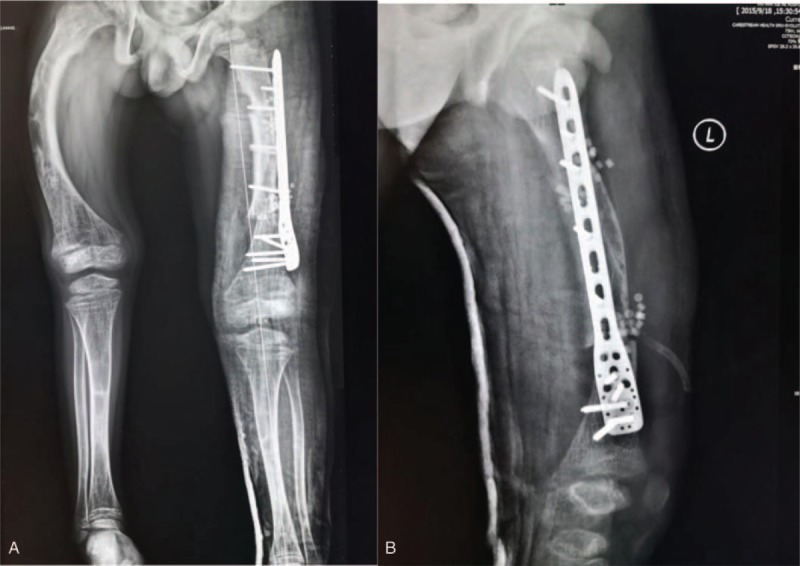
(A) Full-length anterior-posterior view of lower extremity after operation and (B) lateral view of femur after operation.

After the surgery, the malalignment and deviation of was redressed (Fig. 3).

**Figure 4:**
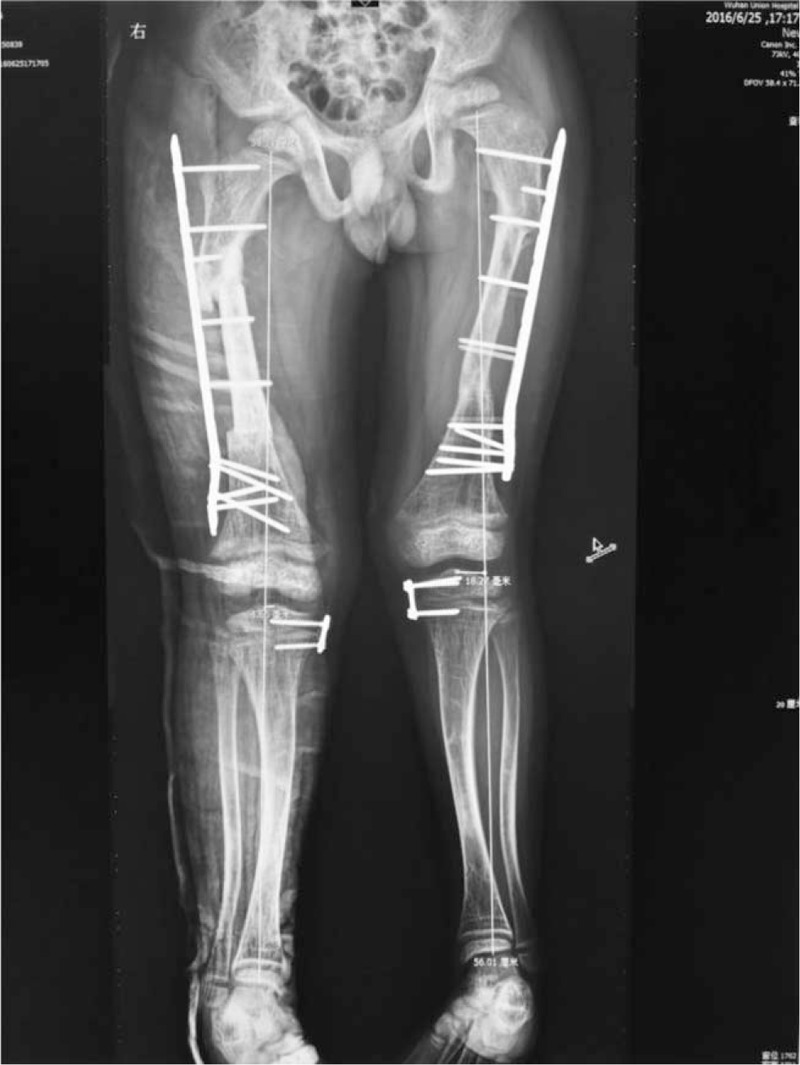
Full-length radiograph of lower extremities after second operation.

Six months later, the same procedures were performed on the right femur, and guided growth (temporary hemiepiphysiodesis) was performed on both tibias (Fig. 4).

**Figure 5:**
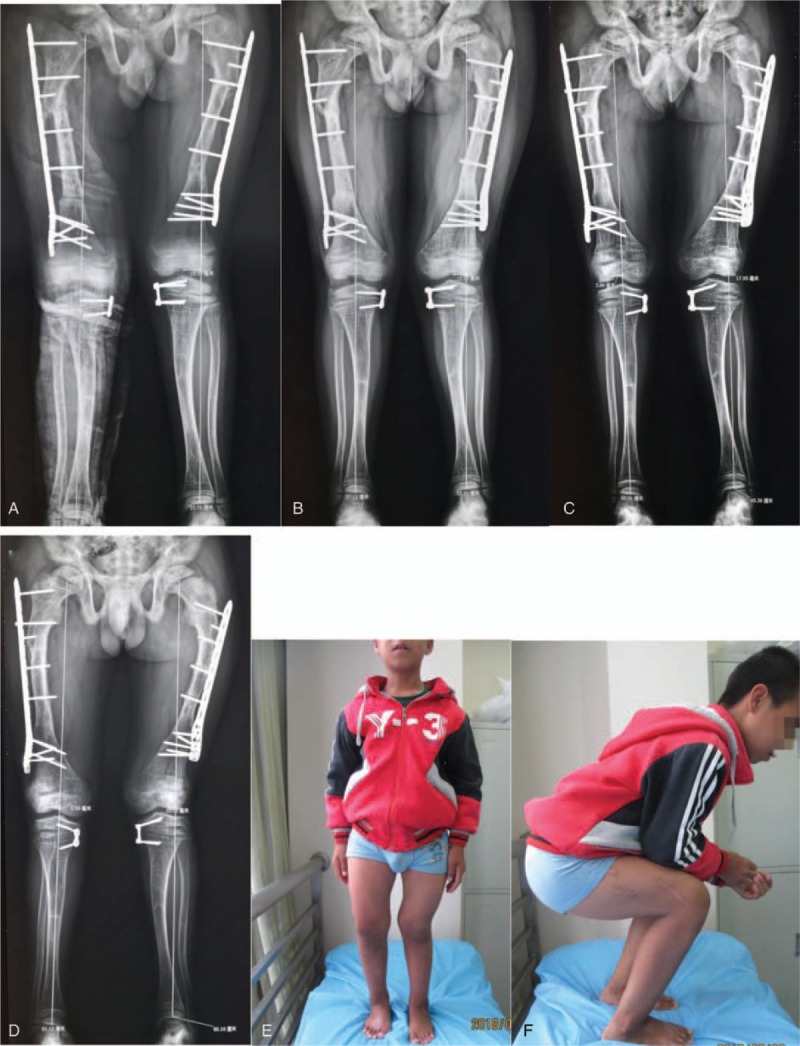
(A) Two months after second operation; (B) 5 months after second operation; (C) 12 months after second operation; (D) 28 months after second operation; (E) appearance of patient on standing position; and (F) flexion of hip and knee joint.

At recent follow-up in August 2018, the boy presented with normal balance and gait, and he was able to stand, walk, and sit almost normally. The mechanical axis was slightly deviated to the lateral side of left knee joint, but the patient was quite satisfied with his current status (Fig. 5).

## 3 Discussion

The XLHR is a metabolic disorder first described by Albright et al in 1937.^[7]^ Patients manifest short stature, reduced growth rate, bone deformities such as coxa vara, femoral and crural bowing, genu varum, and valgum. Early medical treatment has been shown to lead to better results.^[8]^ Medical treatment consists of oral phosphate substitution and supplementation of active vitamin D compounds. Despite correct medical treatment, symptoms such as deformity, pain, short stature can persist, and in some cases, fractures can occur. Over the past 20 years, a wide range of doses between 10 to 80 ng/kg per day of calcitriol and 30 to 180 mg/kg per day of elemental phosphorus prescription has been reported.^[9]^ In this patient, medical treatment was initiated after the diagnosis.

Progressive bowing of the legs will occur with the onset of weight-bearing, and may hinder the walking capacity. Diaphyseal metaphyseal regions of long bones in lower extremities manifest bowing in frontal plan with varus or valgus combined with a degree of flexion. The response of pain and skeletal deformities to medical treatment including adequate doses of calcitriol and phosphorus plus analgesics is good, but the long bone bowing may not be entirely resolved and the pain may persist.^[10,11]^ Surgery is needed for patients with progressive limb deformity. The primary objective of corrective surgery is to readjust the mechanical axis to avoid pain and secondary joint degeneration. It has been suggested that corrective surgery should be postponed until the bone maturation because of the increased risks of nonunion and recurrence of deformity in teenagers.^[12]^ In patients of immature skeleton, there is significant risk of recurrence of the bowing at the level of osteotomy or secondary to the adjacent epiphysiodesis. However, in order to reduce the risk of arthritis and degradation of articular cartilage, the concept in our institution is to do corrective surgery when medical treatment fails to maintain normal mechanical axes and longitudinal growth, independently of age or bone maturation.

About 24% to 65% of patient of HR requires surgical intervention to correct lower limb deformity.^[6]^ In clinical practice, patients with persistent or worsening coronal plane deformity benefit from a corrective operation. Serum phosphate level is always below normal in all patients and is not the predictive factor of the requirement for surgery. However, the serum phosphate level of below 2.5 mg/dL (0.81 mmol/L) to be a predictive factor of worse outcome after surgery.^[13]^ In this case, the serum phosphate level was 1.0 mmol/L; therefore surgery was not contraindicated.

Reported recurrence after corrective surgery is about 0% to 90%,^[14,15]^ and it appears to be related to the timing and fixation method. Patients who received surgery before skeletal maturity generally had a higher recurrence rate.^[14,15]^

Historically speaking, at our medical center, surgery for HR patients consisting of multiple osteotomies, was performed preferably at skeletal maturity. For children aged 5 to 7 years old with an angular deformity on coronal plane, our choice is osteotomy and fixation with K-wires or guided growth technique. For children before skeletal maturation, TSF or Ilizarov was adopted to redress the deformity. In children after skeletal maturity, interlocking nails were preferred over plating.^[12]^ This patient was an 11-years-old teenager about 110 cm in height. The interlocking nail obviously was not a suitable option. Both the TSF and Ilizarov are powerful tools for correcting angular deformities. Compared with the Ilizarov device, TSF allows simultaneous gradual correction of multiplanar deformities and limb lengthening through the osteotomy site.^[16]^ The stability of the circular fixator permits early weight-bearing and provides an ideal environment for new bone formation and soft tissue healing. But TSF and Ilizarov were too bulky and too expensive for this patient from a financially distressed family. Besides, it was challenging to educate the uneducated family members to care for the external fixators meticulously and perform fine-tuning of the alignment postoperatively. Therefore, PHILOS plate was adopted to fixate the osteotomies.

Guided growth using eight plates is a viable option for mild varus or valgus deformity. The adolescent onset varus deformity does not respond well to hemiepiphysiodesis.^[17]^ A slower correction has been observed in patients with metabolic causes, and an earlier start of intervention for patients of idiopathic deformities is indicated.^[18]^ A slight overcorrection of 5° is recommended to achieve the desired effect. Since the patient was an adolescent teenager who was not able to walk normally before surgery, guided growth technique was not suitable for this patient to correct his femoral bowing. In the second surgery, guided growth using 8 plates was adopted to treat the mild valgus deformity of the tibia.

In patients with osteogenesis imperfecta with a severe deformity, multiple osteotomies with the fixation by the telescoping intramedullary nail might be a better option.^[19]^ But in patients of HR, nonunion could be a troublesome complication. Some authors observed an increased rate of nonunion in growing children if medical treatment was insufficient, whereas, in adult patients with a mature skeleton, the healing rate after osteotomy was unremarkable.^[20]^ Therefore, in this patient, rotational and translational technique was used to address this dilemma with subtrochanteric and supracondylar osteotomies (Fig. 2). This technique was derived from the procedures utilized in the correction of scoliosis. The severe bowing at the coronal plane was changed into bowing in sagittal plane. With slight translation of the resected fragment, this bowing degree would decrease to an acceptable realm. At the follow-up, the osteotomy site healed uneventfully, and the patient was back to daily activities and normal sports. In summary, rotational and translational osteotomy is an alternative technique in patient of severe bowing deformity.

## Author contributions

**Data curation:** Pan Hong.

**Formal analysis:** Xin Tang, Ruikang Liu.

**Funding acquisition:** Jin Li.

**Investigation:** Pan Hong.

**Methodology:** Pan Hong.

**Software:** Ruikang Liu.

**Supervision:** Jin Li.

**Validation:** Renhao Ze.

**Writing – original draft:** Pan Hong, Jin Li.

**Writing – review & editing:** Saroj Rai, Jin Li.

Pan Hong orcid: 0000-0003-2674-3016.
